# Retraction: New Criteria Could Improve the Success Rate of Non-operative Management of Acute Appendicitis in Children

**DOI:** 10.7759/cureus.r76

**Published:** 2023-07-21

**Authors:** Osman Uzunlu, Incinur Genisol

**Affiliations:** 1 Pediatric Surgery, School of Medicine, Pamukkale University, Denizli, TUR

This article has been retracted at the request of the authors due to numerous data inaccuracies and discrepancies resulting from flawed database collection between 2016 and 2019 while the database was being used for further research. These errors have a negative effect on the remaining text including the results and conclusions, thus the article has been retracted to correct the scientific record.

